# Present-Biased Preferences Associated with Socioeconomic Status in Intertemporal Decision-Making: Evidence from Behavioral and Electroencephalographic Measures

**DOI:** 10.3390/brainsci16080879

**Published:** 2026-08-18

**Authors:** Haibo Zhou, Yaru Yang, Liangliang Yi, Xinxin Xiang, Yutong Liu

**Affiliations:** School of Education, Hunan University of Science and Technology, Xiangtan 411201, China

**Keywords:** socioeconomic status, intertemporal choice, choice consistency, hierarchical Bayesian modeling, event-related potentials

## Abstract

**Highlights:**

**What are the main findings?**

- The low socioeconomic status (SES) group displayed a higher tendency to prefer immediate rewards during intertemporal decision-making and displayed greater variability in their choices, indicating more random decision behavior.
- The low-SES group exhibited larger P200 amplitudes but smaller N2 and P300 amplitudes. During the later stage of decision-making, P300 amplitudes did not differ significantly between the smaller-sooner (SS) and larger-later (LL) conditions in the low-SES group.

**What are the implications of the main findings?**

- This study provides evidence regarding the cognitive characteristics associated with low SES in individual intertemporal decision-making from behavioral, computational modeling, and electrophysiological perspectives.
- Although the low-SES group showed a greater tendency to choose SS rewards, they did not exhibit a significant P300 compound toward either SS/LL rewards. These findings provide a novel understanding of myopic decision-making under low-SES conditions.

**Abstract:**

**Background**: The role of socioeconomic status (SES) in individual intertemporal decision-making has received widespread research attention. However, findings regarding its underlying psychological mechanisms remain inconclusive. This study investigates not only the association between SES and intertemporal decision-making but also the underlying cognitive neural mechanisms from both behavioral and electrophysiological perspectives. **Methods**: Individuals with high and low SES were recruited. In Experiment 1, a behavioral experiment used the Monetary Choice Questionnaire to measure delay discounting rates using the Kirby method and hierarchical Bayesian modeling, as well as choice consistency using the inverse temperature parameter of the softmax function, to examine differences in decision-making between the two groups. In Experiment 2, event-related potential techniques were employed to examine differences in dynamic neural activity during an intertemporal choice task between the high- and low-SES groups. **Results**: The behavioral results revealed that, in Experiment 1, the low-SES group exhibited a larger delay discounting rate than the high-SES group. The hierarchical Bayesian modeling further indicated that the low-SES group had lower choice consistency. Experiment 2 found that the low-SES group showed a significantly higher proportion of smaller-sooner (SS) choices than the high-SES group. Electroencephalographic results revealed that, compared with the high-SES group, the low-SES group exhibited larger P200 amplitudes but smaller N2 and P300 amplitudes. Within the high-SES group, the larger-later (LL) option elicited more negative N2 and larger P300 amplitudes than the SS option. Conversely, in the low-SES group, P300 amplitudes did not differ significantly between the SS and LL options during the later stage of intertemporal decision-making. **Conclusions**: The results showed that the low-SES group was more inclined toward immediate gains, made more random choices, displayed higher variability, relied more on intuitive heuristic strategies, and exhibited weaker cognitive control and reduced cognitive resource allocation during intertemporal decision-making. However, at the later stage, the absence of a significant difference between SS and LL options provided preliminary EEG evidence consistent with the possibility that individuals in the low-SES group did not completely disregard future outcomes.

## 1. Introduction

Intertemporal choice refers to a decision-making process in which individuals make choices among alternatives whose costs and benefits are experienced at different points in time [1], typically between smaller-sooner (SS) rewards and larger-later (LL) rewards [2,3]. Individuals’ intertemporal choice preferences are closely related to important domains such as consumption, investment, and education [4]. In this decision-making process, the phenomenon whereby the subjective value of future rewards diminishes over time is referred to as delay discounting [5,6]. However, intertemporal choice does not exhibit a uniform pattern across all populations, instead revealing significant individual differences in preferences. This heterogeneity manifests across multiple dimensions, including age development [7], cultural background [8], socioeconomic status (SES) [9], and personality traits [10]. Among these, SES, a key indicator of social stratification, has attracted increasing research attention in recent years [9,11].

SES is a multidimensional construct that reflects not only individuals’ access to material and social resources but also their relative standing in the social hierarchy [12]. Low SES is associated with financial scarcity, environmental instability, and lower subjective social status, among other factors [13]. The existing literature has suggested that SES exerts a profound effect on psychological functioning, influencing an individual’s ability to improve their material circumstances [14,15]. Previous studies have found that low-SES individuals living in poverty tend to exhibit lower saving intentions [16], engage in overborrowing behaviors [17], and make insufficient investments in children’s education [18]. They appear to focus more on satisfying short-term needs while disregarding long-term benefits and returns. These myopic decisions further exacerbate future scarcity, thereby trapping individuals in a cycle in which low SES influences behavioral decisions, which consequently reinforce low SES [19]. Therefore, investigating the intertemporal choice behavior of individuals from different SES backgrounds is of great significance.

Previous findings regarding the association between SES and intertemporal choice, as well as the mechanisms underlying this association, remain inconclusive. Some studies have shown that low-SES individuals exhibit higher delay discounting rates, indicating a stronger tendency to choose immediate rewards [9,11,20,21,22,23]. In this regard, previous studies have indicated that low-SES individuals exhibit poorer cognitive functioning [24], which may further limit their ability to resist temptation and inhibit immediate impulses, thereby affecting their judgment and decision-making [25]. However, several studies have reported no significant effects of SES on intertemporal decision-making [26,27]. In certain contexts, financial scarcity has even been found to increase individuals’ preference for LL rewards [28,29]. Additionally, some scholars have proposed that decision-making under financial scarcity reflects a deliberate and adaptive choice rather than impaired cognitive functioning [28].

Existing studies have proposed different psychological mechanisms, and their inconsistent findings may stem from multiple factors. On the one hand, previous research has largely adopted a correlational approach, focusing on the description of phenomena [9,11], with insufficient exploration of the causal relationships and mechanisms between SES and intertemporal decision-making. On the other hand, this may be attributed to differences in research methods and methodological approaches. Differences across studies in the dimensions of amount and time may lead to discrepancies in the fitting of results. Given these challenges, standardized and well-validated research tools should be employed to investigate the underlying mechanisms, especially the cognitive neural mechanisms, through which SES influences intertemporal decision-making from a causal perspective. Among the available assessment tools, the Monetary Choice Questionnaire (MCQ), originally introduced by Kirby et al. [30], has been widely adopted worldwide because of its strong psychometric properties [6,31,32]. This facilitates cross-cultural comparisons and promotes research replicability. In terms of exploring underlying mechanisms, event-related potential (ERP), characterized by excellent temporal resolution, has been extensively applied in studies related to SES [33,34,35] and intertemporal decision-making [36,37,38,39]. By examining the temporal dynamics of intertemporal decision-making processes, in-depth investigations can be conducted into cognitive neural mechanisms associated with SES, including cognitive control [40,41,42,43] and attentional resources [44].

In summary, this study aims to examine how SES is associated with intertemporal choice through two experiments. Experiment 1 consists of a behavioral experiment in which participants from high- and low-SES groups are recruited, and their delay discounting behavior is measured using the MCQ [30]. A higher delay discount rate indicates a stronger inclination toward immediate rewards, reflecting myopic decision-making. Recently, increasing attention has been paid to choice consistency [45,46,47,48] also referred to by some researchers as choice stochasticity [49] or choice sharpness [6]. Choice consistency refers to the extent to which participants consistently choose the option with the higher subjective value throughout the intertemporal choice task [50].

Experiment 2 employs ERP techniques to explore the cognitive neural mechanisms underlying intertemporal choice in high- and low-SES groups. Regarding electrophysiological activity in intertemporal decision-making, previous research has mainly focused on the dynamic neural responses of the P200, N2, and P300 components. The P200 component has been associated with early attentional allocation, feature detection and encoding, emotional salience processing, and stimulus evaluation during cognitive processing [51,52,53,54]. Previous studies have suggested that an increased P200 amplitude has been linked to greater reliance on intuitive heuristic strategies during the early recognition and evaluation of stimuli [55], particularly in the context of intertemporal decision-making [37,56]. The N2 component has been associated with early selective attention, conflict monitoring, and cognitive control during decision-making processes [57,58,59]. In delay discounting tasks, the N2 component has been associated with individuals’ ability to resist immediate rewards [52,60]. More negative N2 amplitudes have been linked to greater engagement of cognitive control resources during intertemporal decision-making [59,60]. The P300 component is generally considered to be associated with attentional processing, stimulus evaluation, decision-making, and memory processes [61]. For intertemporal choice, the P300 component tends to reflect allocation of attentional resources during the later stages of decision-making [62,63]. When individuals engage greater decision-related cognitive resources, including attentional resources, stronger P300 activity is elicited [64].

## 2. Experiment 1

### 2.1. Methods

#### 2.1.1. Participants

G-Power (3.1.9.7) was employed to determine the minimum required sample size [65]. Using α = 0.05 and a statistical power of 0.80, the analysis indicated that a minimum sample of 128 participants was required to detect a medium effect size (Cohen’s *d* = 0.50). Participants were recruited from four universities in Xiangtan City, Hunan Province, China, via the Credamo platform [66,67]. Given that SES is a multidimensional construct encompassing educational attainment, economic income, occupational status, social mobility, and access to resources, no consensus has been reached regarding a standardized approach for its assessment. Accordingly, the present study established participant recruitment criteria based on previous research, which has demonstrated applicability in the Chinese context [68]. The inclusion criteria for the low-SES group were as follows: (1) a monthly per capita income of less than 1000 CNY, (2) coming from poverty-stricken households officially identified by the school, and (3) having applied for student loans. The inclusion criteria for the high-SES group were as follows: (1) a monthly per capita income exceeding 10,000 CNY and (2) not fulfilling any of the conditions specified for the low-SES group. Ultimately, this study recruited 110 participants for the low-SES group (20.31 ± 1.61 years, 70 females) and 110 participants for the high-SES group (20.68 ± 1.59 years, 65 females). All participants were right-handed, had normal or corrected-to-normal vision, and reported no history of neurological or affective disorders. They were familiar with computer operations and had no previous experience with comparable experiments. Upon completion of the experiment, participants received financial compensation for their participation. Before participating in the study, all participants provided written informed consent and were informed that they could withdraw at any time. Ethical approval for the study was granted by the Ethics Committee of the School of Education, Hunan University of Science and Technology, in accordance with the principles of the Declaration of Helsinki and its subsequent amendments (No. 2025-10).

#### 2.1.2. Materials

Subjective SES. Participants’ subjective SES was assessed using the MacArthur Scale [69], which has been widely employed in prior studies [70,71,72]. Participants were presented with an illustration of a 10-rung ladder, with rungs ranging from 1 (lowest) to 10 (highest). They were instructed to indicate the rung that best reflected their perceived standing within the social hierarchy relative to others, based on their income, education, and occupational status. Higher scores indicate higher perceived SES.

Objective SES. To examine differences between the two groups in objective SES, this study considered the education levels and occupational status of the participants’ parents, based on previous research [12,73,74]. Occupational status was scored on a 10-point scale (1 = unemployed, 2 = agricultural laborer, 3 = industrial worker, 4 = commercial service personnel, 5 = self-employed individual, 6 = administrative staff, 7 = professional and technical personnel, 8 = private enterprise owner, 9 = managerial personnel, 10 = state and social administrators) [12]. Parental educational attainment was assessed using a five-point scale (1 = no education, 2 = primary school, 3 = secondary school, 4 = postsecondary education, 5 = university) [73].

Intertemporal Choice Task. The MCQ [30] was adopted to measure participants’ delay discounting tendencies because it has demonstrated high test–retest reliability and has been widely employed owing to its strong research utility [6,31,32]. The original currency unit of the questionnaire was U.S. dollars. Following prior research [75], this study converted the currency unit into CNY while keeping the monetary amounts and time intervals unchanged. The task comprised 27 trials (see Supplementary Materials, Table S1), during each of which participants were asked to choose between an SS and an LL reward.

#### 2.1.3. Experiment Design and Procedure

Experiment 1 employed a between-subjects design with one factor and two levels (SES: low vs. high). The dependent variables included the delay discounting rate (Kirby method and hierarchical Bayesian models) and choice consistency (inverse temperature parameter of the softmax function), both of which were measured during the intertemporal choice task.

**Figure 1:**
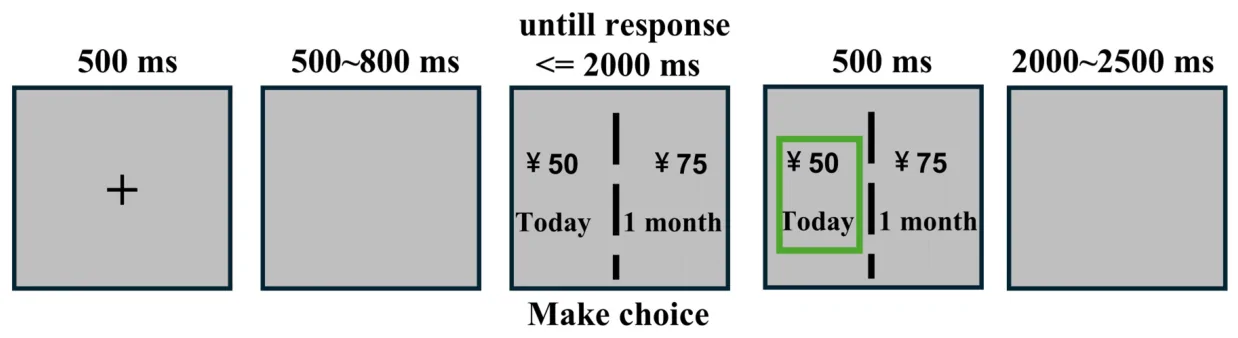
Intertemporal choice task flowchart. In each trial, two options (SS and LL) are simultaneously and randomly displayed on the left and right sides of the screen, remaining balanced throughout the experiment. Participants selected the left or right option using the “F” or “J” key, respectively. After the participant’s response, a green boxes indicates the selected response. Note: The intertemporal choice task flowchart is identical for Experiment 1 and Experiment 2, although the specific timing and monetary amounts differ.

All participants performed the intertemporal choice task using a computer. The task was presented, and behavioral data were collected using PsychoPy 3 software [76]. The procedure for a single trial is depicted in Figure 1. First, a black fixation point “+” was displayed at the center of the screen for 500 ms, after which a blank screen was presented for a randomly varying duration of 500–800 ms. Subsequently, the smaller-sooner (SS) and larger-later (LL) options were simultaneously presented on the left or right side of the screen. Participants selected their preferred option by pressing a key within 2000 ms (the “F” and “J” keys corresponded to the left and right options, respectively). If participants failed to respond within the specified time, a prompt was displayed, and the trial automatically proceeded to the next one. Once a response was made, a green frame appeared around the chosen option for 500 ms, after which a blank screen was presented for 2000–2500 ms before the next trial began. Prior to the formal experiment, participants completed two practice trials randomly drawn from the formal task to familiarize themselves with the procedure.

#### 2.1.4. Data Recording and Analysis

To verify the effectiveness of this grouping method, independent-samples *t*-tests were conducted to compare the scores obtained from the MacArthur Scale and the Objective SES Scale between the two groups. Specifically, in the latter, each participant’s objective SES score was derived by averaging the educational and occupational scores of both parents to obtain family-level education and occupation scores, respectively. These two scores were subsequently standardized as Z-scores and combined to generate an overall objective SES score [12,77].

Data collected from the intertemporal choice task were processed according to the hyperbolic discounting equation as presented in Equation (1):

(1) $$SV\left({LL}_{t}\right)=\frac{A_{t}}{\left(1+k*D_{t}\right)}$$

Here, SV(LL) represents the subjective value of the LL reward, whereas A and D denote the reward amount and delay, respectively. The parameter (*k*) captures the degree of temporal discounting, with higher values reflecting a steeper decline in subjective value as the reward delay increases.

Following the method proposed by Kirby [30] and related calculation tools [78], this study computed the delay discounting rate for each participant. The specific procedure was as follows. First, the 27 items were arranged according to their corresponding k values in ascending order. Then, each participant’s delay discounting rate was determined based on the switching point among the sorted items. For example, item 5 has an indifference k value of 0.041. At this value, the two rewards are considered equivalent for a participant. Thus, selecting the immediate reward in this trial indicates that the participant’s discount rate is greater than 0.041, whereas selecting the delayed reward indicates that the participant’s discount rate is lower than 0.041. Finally, the geometric mean of the indifference k values of the two items at which the participant’s choice shifted was taken as the participant’s delay discounting rate. If participants’ choices were not entirely consistent, resulting in multiple switching points, the k value with the highest consistency score (i.e., the one best predicting the participant’s preference) was selected as the final delay discounting rate. When the highest consistency score occurred more than once, the final k value was determined by calculating the geometric mean of the k values corresponding to those items. Temporal discount rates were natural log (ln-)transformed to ensure that they followed a normal distribution and were referred to as ln(*k*) [30,78]. The ln(*k*) values were compared across the high- and low-SES groups using independent-samples *t*-tests.

Meanwhile, this study employed a cognitive modeling approach to analyze the MCQ data and examine differences between the high- and low-SES groups. The discounting rate and choice consistency for each participant were estimated using hierarchical Bayesian modeling to fit a hyperbolic discounting model [6,79], as shown in Equation (2).

(2) $$SV({LL}_{t})=\frac{A_{t}}{\left(1+\exp\left(k\right)*D_{t}\right)}$$

where A_t_ and D_t_ denote the amount and delay of the LL option on trial *t*, respectively.

The hyperbolic model was then combined with the softmax action selection rule. The probability of choosing the LL option on trial *t* is defined in Equation (3):

(3) $$P\left({LL}_{t}\right)=\frac{exp({SV}_{{LL}_{t}}*beta)}{\exp\left({SV}_{{SS}_{t}}*beta\right)+exp({SV}_{{LL}_{t}}*beta)}$$

Here, the parameter *beta* captures the extent to which choices are driven by the valuation model. In decision neuroscience, the behavioral parameter beta, or inverse temperature, describes the choice consistency or stochasticity of individual decisions [6,79]. Given the substantial positive skew typically observed in the distribution of both discount rates and choice consistency, the parameters were estimated as ln(*k*) and ln(*beta*). This transformation mitigates numerical instability near zero and facilitates more reliable parameter estimation [6,30,78].

The softmax function parameters were estimated through the Markov chain Monte Carlo simulation conducted in R (version 4.4.3) [80] using the RStan package (version 2.32.7) [81]. The sampling procedure involved 4000 iterations across 4 chains, with 2000 warmup samples. Chain convergence was assessed via the Gelman–Rubin diagnostic (R-hat), and values below 1.01 were considered acceptable ([82,83]). In hierarchical Bayesian analysis, prior distributions for model parameters are updated based on the observed data to yield posterior distributions, representing the updated beliefs about the parameters conditional on the data. We therefore specified noninformative flat priors to minimize the potential influence of prior assumptions on the posterior distributions. Differences in model parameters between groups were evaluated for two parameters (discounting rate and choice consistency) by estimating the 95% highest density interval (HDI) of the posterior differences between groups, providing a reliable interval estimate of the posterior differences for each parameter. A 95% HDI that did not contain zero indicated a credible difference in the posterior values between the two groups for that parameter [6,46,48,84].

### 2.2. Results

Manipulation checks. The independent-samples *t*-test revealed that the high-SES group (6.25 ± 1.96) scored significantly higher than the low-SES group (4.32 ± 1.74) on the subjective SES scale, *t* (218) = −7.71, *p* < 0.001, Cohen’s *d* = −1.04, 95% CI [−1.32, −0.76]. Similarly, on the objective socioeconomic status scale, the high-SES group (2.55 ± 1.16) scored significantly higher than the low-SES group (−1.35 ± 0.53), *t* (218) = −32.11, *p* < 0.001, Cohen’s *d* = −4.33, 95% CI [−4.81, −3.84].

**Figure 2:**
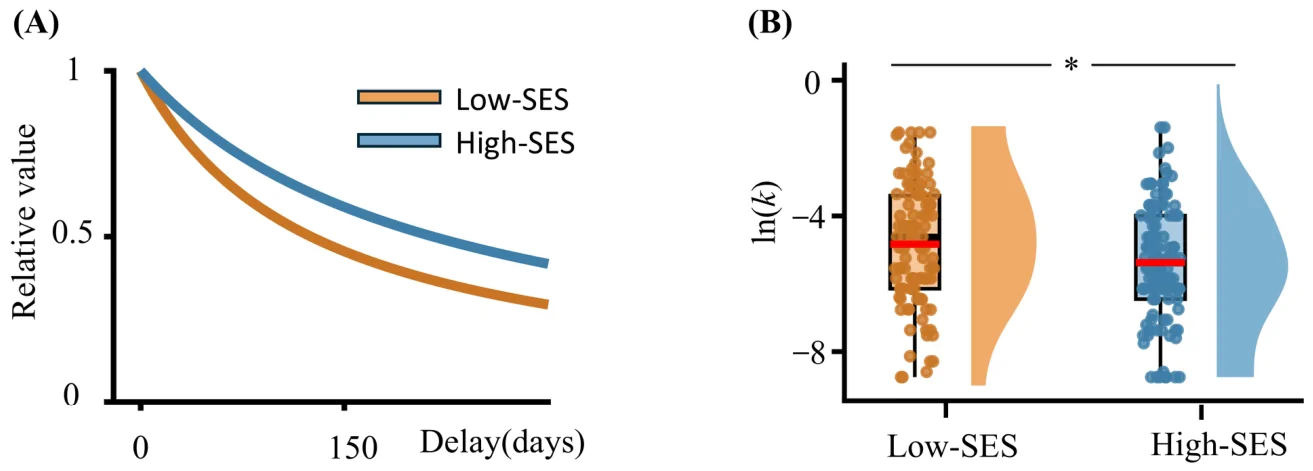
Model-free results based on Kirby’s method. Panel (**A**) presents the hyperbolic discounting curves for each group. Panel (**B**) depicts the ln(*k*) value calculated based on the Kirby method. The red horizontal line indicates the mean value. “Low-SES” indicates the low-socioeconomic-status group, and “High-SES” indicates the high-socioeconomic-status group. * *p* < 0.05.

Model-free and Model Results. The results of the independent-samples *t*-test revealed that the ln(*k*) of the low-SES group (−4.83 ± 1.76) was greater than that of the high-SES group (−5.37 ± 1.73), *t* (218) = 2.29, *p* = 0.023, Cohen’s *d* = 0.31, 95% CI [0.04, 0.57]. This finding indicates that the low-SES group exhibited a higher delay discounting rate and was more inclined to choose smaller, immediate rewards (Figure 2).

**Figure 3:**
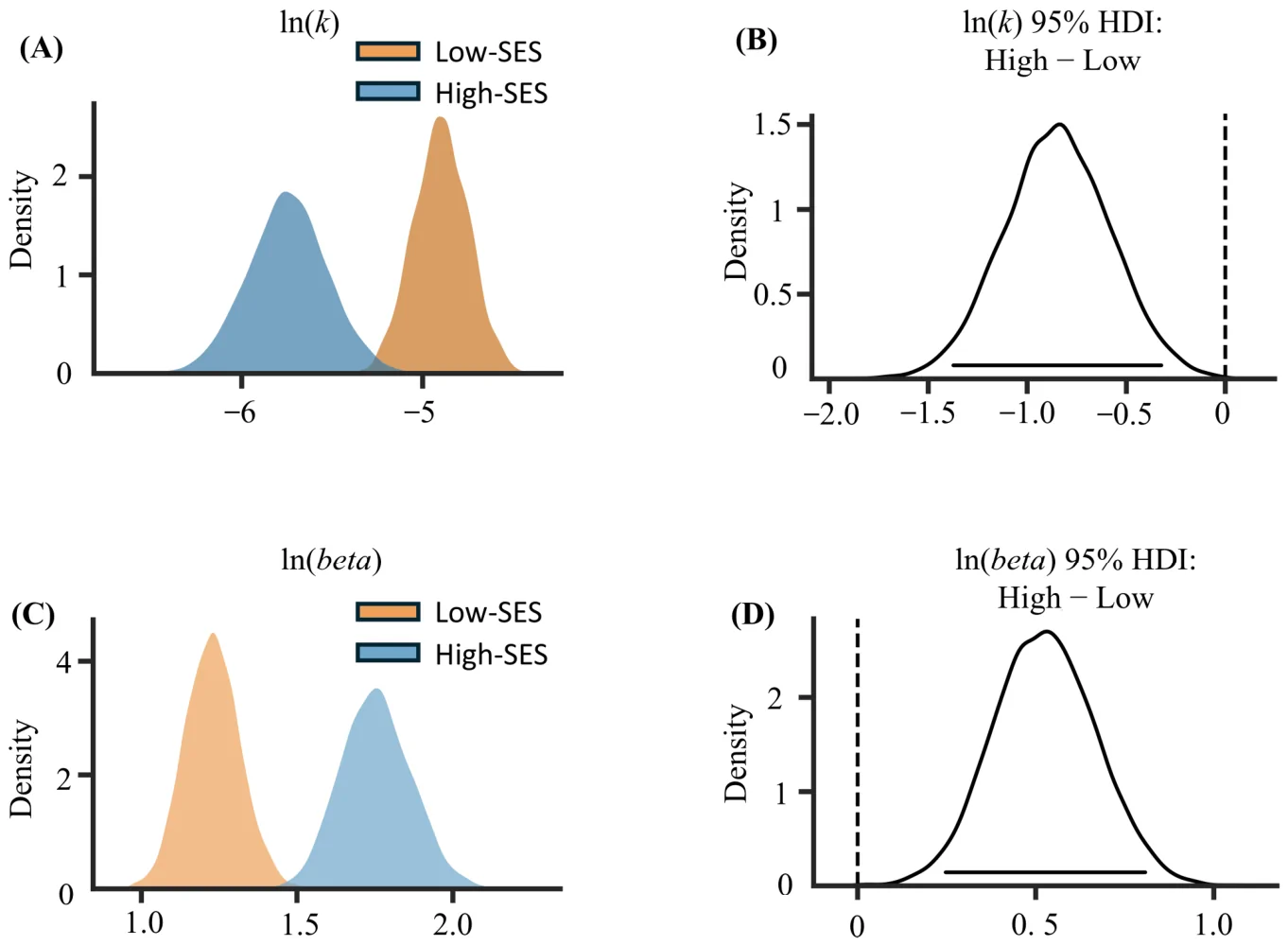
Modeling results from a hierarchical Bayesian model. Specifically, Panels (**A**,**C**) depict the posterior distributions of the discounting rate (ln(*k*)) and the inverse temperature parameter (ln(*beta*)) from the hierarchical Bayesian model for the low-SES group and the high-SES group, respectively. Panels (**B**,**D**) show the posterior density plots of the group differences in discounting rate (ln(*k*)) and choice consistency (ln(*beta*)) between the low-SES and high-SES groups. In panels B and D, the horizontal solid line indicates the 95% HDI, and the vertical dashed line indicates x = 0. The 95% HDI represents the most plausible range of values for the group differences. “Low-SES” indicates the low-socioeconomic-status group, and “High-SES” indicates the high-socioeconomic-status group. “High – Low” indicates the high-SES group minus the low-SES group.

In the hierarchical Bayesian analysis, the posterior group differences were calculated as High SES − Low SES (Figure 3). The low-SES group exhibited a credibly higher ln(*k*) value (HDI_median_ = −0.85, HDI_95%_ = [−1.37, −0.32]) and a credibly lower ln(*beta*) value (HDI_median_ = 0.53, HDI_95%_ = [0.25, 0.81]) than the high-SES group. For both parameters, the HDIs excluded zero, thereby providing credible evidence for larger temporal discounting and lower choice consistency in the low-SES group compared to the high-SES group. Supplementary Material S3 provides additional supporting evidence for the between-group differences identified in the Bayesian analysis of Experiment 1.

### 2.3. Discussion

Experiment 1 employed a behavioral experiment to investigate the association between SES and intertemporal choice. The findings revealed that the ln(*k*) value for the low-SES group was higher than that for the high-SES group, indicating that, compared with the high-SES group, the low-SES group exhibited a higher delay discounting rate and a greater propensity for SS rewards, consistent with previous research findings [20,21]. Furthermore, hierarchical Bayesian modeling revealed that the low-SES group had significantly lower choice consistency than the high-SES group. The findings from Experiment 1 provide causal evidence for the influence of SES on intertemporal decision-making. However, investigation into the cognitive neural mechanisms underlying the influence of SES on intertemporal choice is lacking in the existing research. Therefore, Experiment 2 employed event-related potential techniques to reveal these underlying cognitive neural mechanisms.

## 3. Experiment 2

### 3.1. Methods

#### 3.1.1. Participants

Participants were recruited from four universities in Xiangtan City, Hunan Province, China, via the Credamo platform [66,67]. The recruitment criteria for the high-SES group and the low-SES group were the same as those used in Experiment 1. Ultimately, this study recruited 28 participants for the low-SES group (21.21 ± 1.45 years, 17 females) and 28 participants for the high-SES group (20.79 ± 1.47 years, 16 females). A post hoc sensitivity analysis was performed using G*Power (Version 3.1.9.7). Assuming α = 0.05 and a desired statistical power of 0.80, the final sample size (*N* = 56) provided sufficient power to detect an interaction effect of at least Cohen’s *f* = 0.19 in the repeated-measures ANOVA with a within-between interaction. Participants were right-handed, had normal or corrected-to-normal vision, and reported no history of neurological diseases or affective disorders. They were proficient in using computers and had not previously participated in similar experiments. After completing the experiment, they received remuneration for their participation. Prior to participation, written informed consent was obtained from all participants, who were informed of their right to discontinue the study at any point. This study was reviewed and approved by the Ethics Committee of the School of Education at Hunan University of Science and Technology in accordance with the Declaration of Helsinki and its subsequent amendments (No. 2025-10).

#### 3.1.2. Materials

Subjective and Objective SES.

Same as in Experiment 1.

Intertemporal Choice Task. The task procedure was adapted from the classic intertemporal choice experimental paradigm, with timing and monetary parameters based on previous research [2,3,85,86]. Hypothetical monetary rewards were used as the stimulus materials. Participants were required to choose between SS and LL options. The SS option’s reward was always received “today,” while the LL option’s reward was received after “1 month” or “2 months.” The monetary value (in CNY) for the SS option was randomly drawn from a Gaussian distribution (50 ± 25) and converted into an integer, resulting in values between 13 and 120. The amount of the LL option was increased proportionally relative to the SS option, with percentage differences between the two options set at 5%, 10%, 15%, 25%, 35%, and 50%.

#### 3.1.3. Experiment Design and Procedure

Experiment 2 employed a 2 (SES: low vs. high) × 2 (SS/LL rewards: SS vs. LL) mixed design. The between-subjects variable was SES, and the within-subjects variable was SS/LL rewards. The dependent variables included the proportion of SS choices and reaction time, whereas the electroencephalographical measures included the amplitudes of the P200, N2, and P300 components.

All participants completed the intertemporal choice task in an EEG laboratory equipped with soundproofing and electromagnetic shielding. The stimuli were presented using PsychoPy 3 [76]. The experimental procedure for a single trial is shown in Figure 1. First, a black “+” fixation point was displayed at the center of the screen for 500 ms, followed by a blank screen presented for a randomly determined duration of 500–800 ms. Subsequently, the SS and LL options were simultaneously displayed on the left and right sides of the screen. Participants were required to choose between the two options by pressing the “F” and “J” keys to select the option on the left and right, respectively. Once a response was made, the selected option was immediately surrounded by a green frame, which remained visible for 500 ms. If no key was selected within 2000 ms, the participant received a prompt, and the trial automatically proceeded to the next one. Finally, a blank screen was presented for 2000–2500 ms, after which the next trial began. The experiment comprised two parts: a practice phase and a formal phase. Before the formal experiment began, 8 practice trials were allowed to familiarize participants with the procedure. Once participants fully understood the experimental tasks and could complete them independently and proficiently, they could proceed to the formal experiment, which consisted of four blocks with 80 trials per block, resulting in a total of 320 trials in total. Following the completion of each block, participants could choose to take a break lasting approximately 2 min. The SS/LL rewards were randomly assigned to the left or right side of the screen across trials.

#### 3.1.4. Data Recording and Analysis

Behavioral Analyses

An independent-samples *t*-test was conducted to examine differences in MacArthur Scale scores and objective SES measurements (calculated using the same procedure described in Experiment 1) between the high- and low-SES groups, thereby validating the grouping procedure. Behavioral data recorded during the intertemporal choice task were processed. The proportion of choosing the SS option was calculated for the high- and low-SES groups, followed by an independent-samples *t*-test. A two-factor repeated measures analysis of variance was conducted with SES and SS/LL rewards as the independent variables and reaction time during the intertemporal choice task as the dependent variable.

EEG Recording and Analyses

This study recorded EEG signals using Scan 4.5 software (NeuroScan Inc., Charlotte, NC, USA) with a 64-channel electrode cap configured according to the extended version of the International 10–20 System. During data recording, the online reference electrode was located at the midpoint between Cz and CPz. In addition, two electrodes were placed 1 cm above and below the right eye to record vertical electrooculography (VEOG), and one electrode was placed at the outer canthus of each eye to record horizontal electrooculography (HEOG). Furthermore, EEG recordings were processed with a 0.01–100 Hz bandpass filter, with data acquired at a sampling rate of 500 Hz. Electrode impedances remained below 5 kΩ throughout data collection.

After data collection had been completed, the EEG data were analyzed offline using EEGLAB [87]. During offline data processing, EEG signals were re-referenced to the mean activity of all scalp electrodes. Noisy channels were identified and interpolated using a spherical spline interpolation. Subsequently, continuous EEG data were decomposed via independent component analysis in EEGLAB. Components corresponding to eyeblinks and bodily movements were isolated and discarded with the aid of the ICLabel algorithm [88]. Furthermore, ERPLAB [89] was used for subsequent analysis of the EEG data. EEG data were segmented into epochs spanning from 200 ms prior to stimulus onset to 1000 ms following stimulus onset, with the 200 ms prestimulus period serving as the baseline for correction. To eliminate interference from electrooculographic and motion artifacts, trials with voltages exceeding ±100 μV were excluded. Data from all participants were included in the final analysis. The number of valid trials retained for each condition was as follows: low-SES-SS condition, 44 ± 7; low-SES-LL condition, 43 ± 6; high-SES-SS condition, 41 ± 6; and high-SES-LL condition, 42 ± 6. The four conditions were combined and averaged to obtain the final average EEG waveforms.

Guided by the grand-averaged ERP waveforms and findings from previous research [36], mean amplitude analyses were conducted for three ERP components (P200, N2, and P300). The temporal windows for analyzing the P200, N2, and P300 components were specified by integrating the prior literature with visual inspection of the grand-averaged waveforms across all conditions (collapsed across experimental factors to avoid bias). Candidate latency ranges were initially bounded according to previous intertemporal decision-making studies [36,52,53,59,60,63,90]. Next, the exact peak latencies were visually identified from the grand-averaged waveforms, and symmetrical time windows centered around these peak latencies were established for statistical analysis. For the P200 component, the time window was set to 160–210 ms, and analyses were performed on electrodes Fz, F3, and F4. The time window for the N2 component was set to 240–290 ms, with analysis conducted at electrodes Fz, F3, and F4. The time window for the P300 component was set at 300–420 ms, with analysis conducted at electrodes Pz, P3, and P4. A 2 (SES: low vs. high) × 2 (intertemporal choice: SS option vs. LL option) repeated-measures analysis of variance (ANOVA) was conducted with socioeconomic status (SES) as the between-subjects factor and intertemporal choice as the within-subjects factor. The Greenhouse–Geisser correction was applied to all *p*-values obtained from the ANOVAs. Additionally, the Bonferroni correction was applied to control for multiple comparisons.

### 3.2. Results

Manipulation checks. The independent-samples *t*-test revealed that the high-SES group (6.36 ± 1.54) scored significantly higher than the low-SES group (4.29 ± 1.33) on the subjective SES scale, *t* (54) = −5.38, *p* < 0.001, Cohen’s *d* = −1.44, 95% CI [−2.02, −0.84]. Similarly, on the objective SES scale, the high-SES group (2.47 ± 0.98) scored significantly higher than the low-SES group (−1.32 ± 0.41), *t* (54) = −18.88, *p* < 0.001, Cohen’s *d* = −5.05, 95% CI [−6.12, −3.96].

#### 3.2.1. Behavioral Results

The proportion of SS choices

The independent-samples *t*-test revealed that the proportion of SS choices was higher in the low-SES group (0.58 ± 0.10) than in the high-SES group (0.53 ± 0.08), *t* (54) = 2.13, *p* = 0.037, Cohen’s *d* = 0.57, 95% CI [0.03, 1.10].

Reaction time

For the reaction time, repeated-measures ANOVA showed a significant main effect of SES [F (1, 54) = 7.69, *p* = 0.008, $\eta_{p}^{2}$ = 0.125]. Specifically, participants in the low-SES group exhibited significantly longer reaction times (878 ± 262 ms) than those in the high-SES group (776 ± 191 ms). The main effect of SS/LL rewards was not significant, and neither was the interaction between SES and SS/LL rewards (all *p* > 0.05).

#### 3.2.2. ERP Results

P200

**Figure 4:**
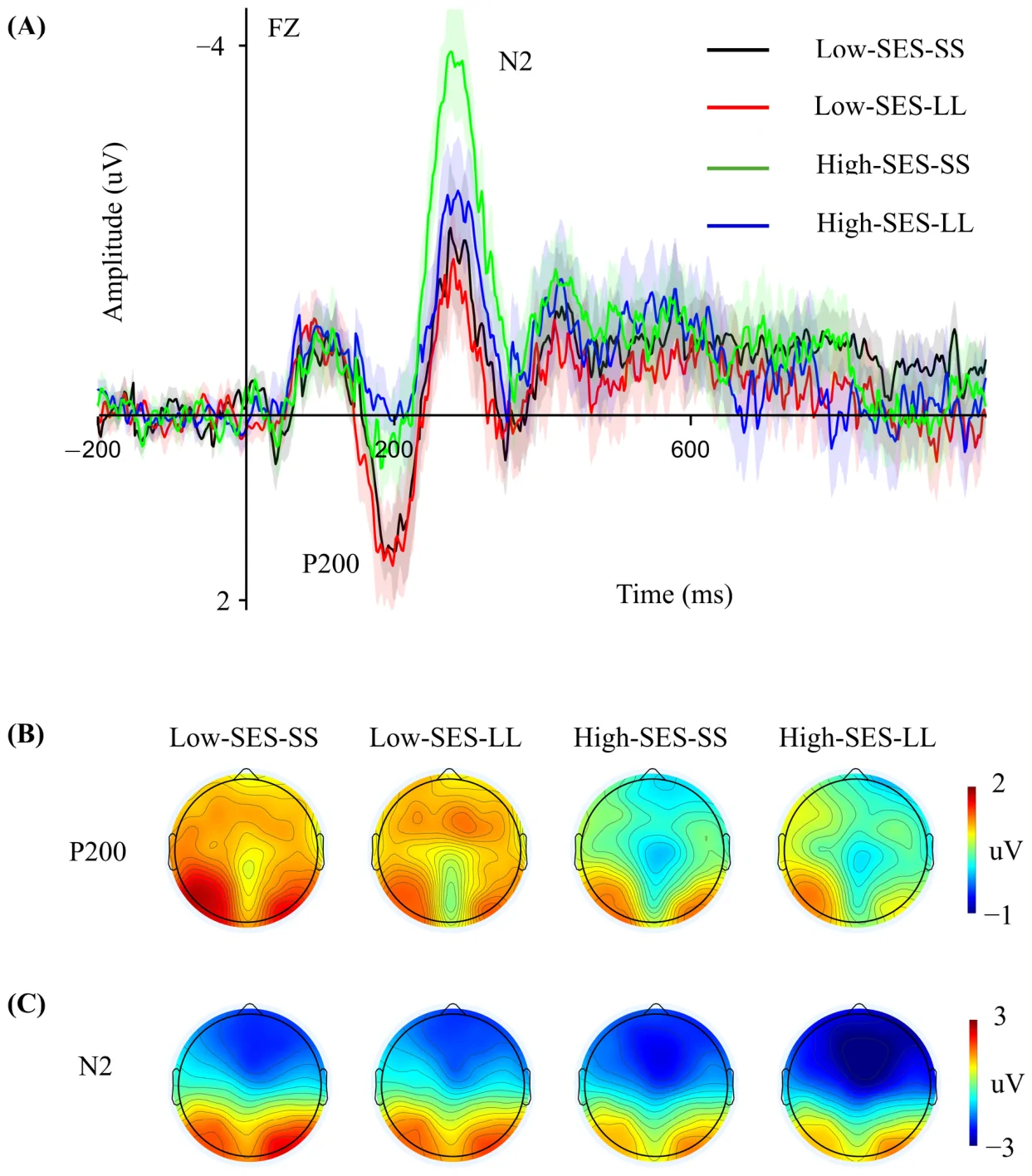
Grand-averaged ERP waveforms of the P200 and N2 components (no additional filtering applied). Panel (**A**) depicts the grand-averaged waveforms recorded from the Fz electrode. Panel (**B**) and (**C**) illustrates the scalp topographies of the P200 and N2 components. “Low-SES” indicates the low-SES group, and “High-SES” indicates the high-SES group. “SS”, smaller-sooner; “LL”, larger-later.

**Figure 5:**
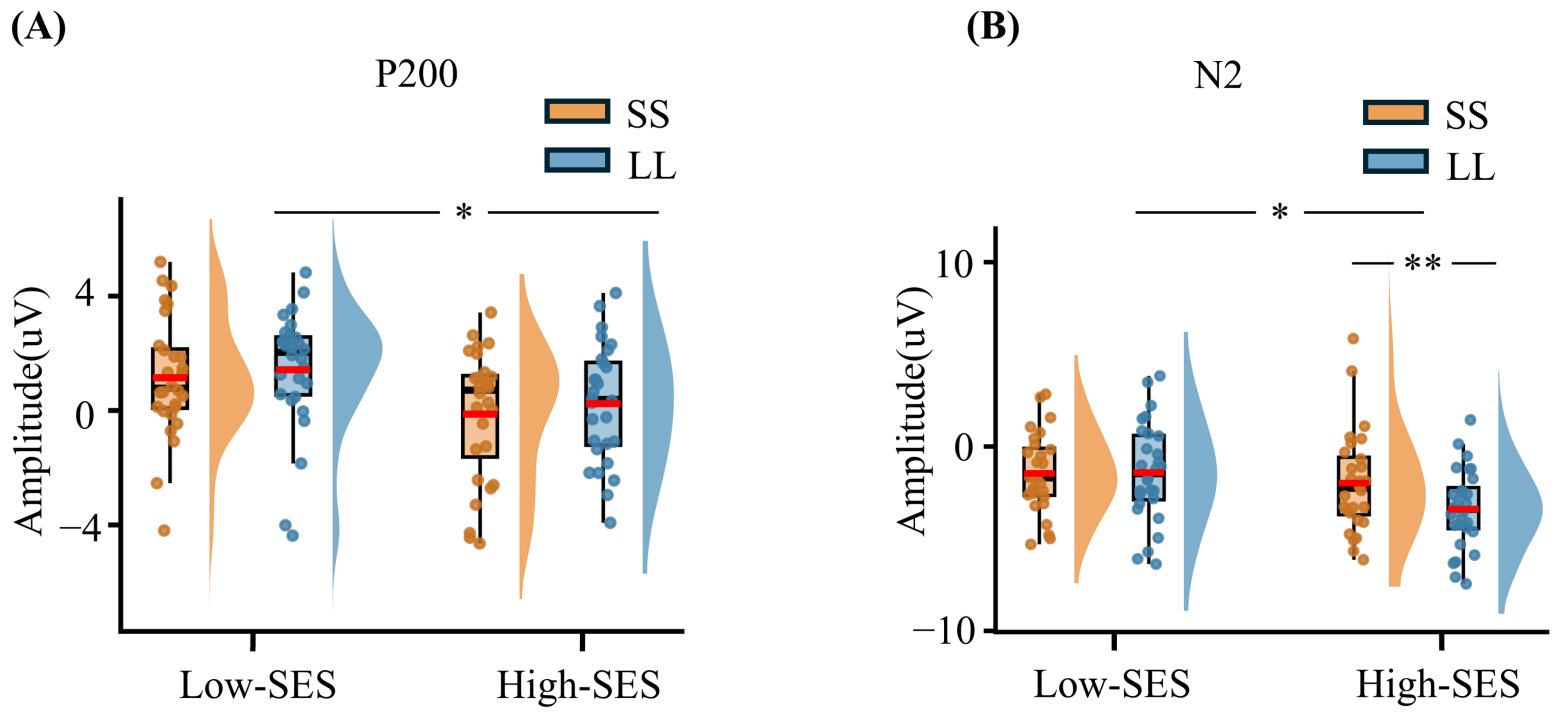
Mean amplitude of the P200 and N2 components. Panel (**A**) shows the average amplitude of P200, and Panel (**B**) shows the average amplitude of N2. The red horizontal line indicates the mean value. “Low-SES” indicates the low-SES group, and “High-SES” indicates the high-SES group. “SS”, smaller-sooner; “LL”, larger-later. * *p* < 0.05, ** *p* < 0.01.

For the P200 amplitude, the repeated-measures ANOVA revealed a significant main effect of SES [F (1, 54) = 5.40, *p* = 0.024, $\eta_{p}^{2}$ = 0.091]. The low-SES group exhibited a significantly more positive P200 amplitude than the high-SES group. No other significant main effects or interactions were detected (all *p* > 0.05) (Figure 4 and Figure 5; additional details are presented in the Supplementary Materials, Table S2).

N2

For the N2 amplitude, repeated-measures ANOVA showed a significant main effect of SES [F (1, 54) = 4.51, *p* = 0.038, $\eta_{p}^{2}$ = 0.077], with the high-SES group exhibiting more negative N2 amplitudes than the low-SES group. The main effect of SS/LL rewards was also significant [F (1, 54) = 5.38, *p* = 0.024, $\eta_{p}^{2}$ = 0.091], indicating that the N2 amplitude elicited by the LL option was significantly more negative than that elicited by the SS option (−1.72 ± 0.33 μV). The interaction between SES and SS/LL rewards was also significant [F (1, 54) = 6.13, *p* = 0.016, $\eta_{p}^{2}$ = 0.102]. Simple-effects analysis indicated that, in the low-SES group, the N2 amplitude did not differ significantly between the LL option and the SS option [−1.46 ± 0.47 μV; F (1, 54) = 0.01, *p* = 0.913, $\eta_{p}^{2}$ = 0.001]. In the high-SES group, the N2 amplitude was significantly more negative in the LL option than in the SS option, F (1, 54) = 11.49, *p* = 0.001, $\eta_{p}^{2}$ = 0.175. For the SS option, no significant difference in N2 amplitude was found between the high-SES group and the low-SES group, F (1, 54) = 0.62, *p* = 0.434, $\eta_{p}^{2}$ = 0.011. For the LL option, the N2 amplitude was significantly more negative for the high-SES group than in the low-SES group, F (1, 54) = 9.17, *p* = 0.004, $\eta_{p}^{2}$ = 0.145 (Figure 4 and Figure 5; additional details are presented in the Supplementary Materials, Table S2).

P300

**Figure 6:**
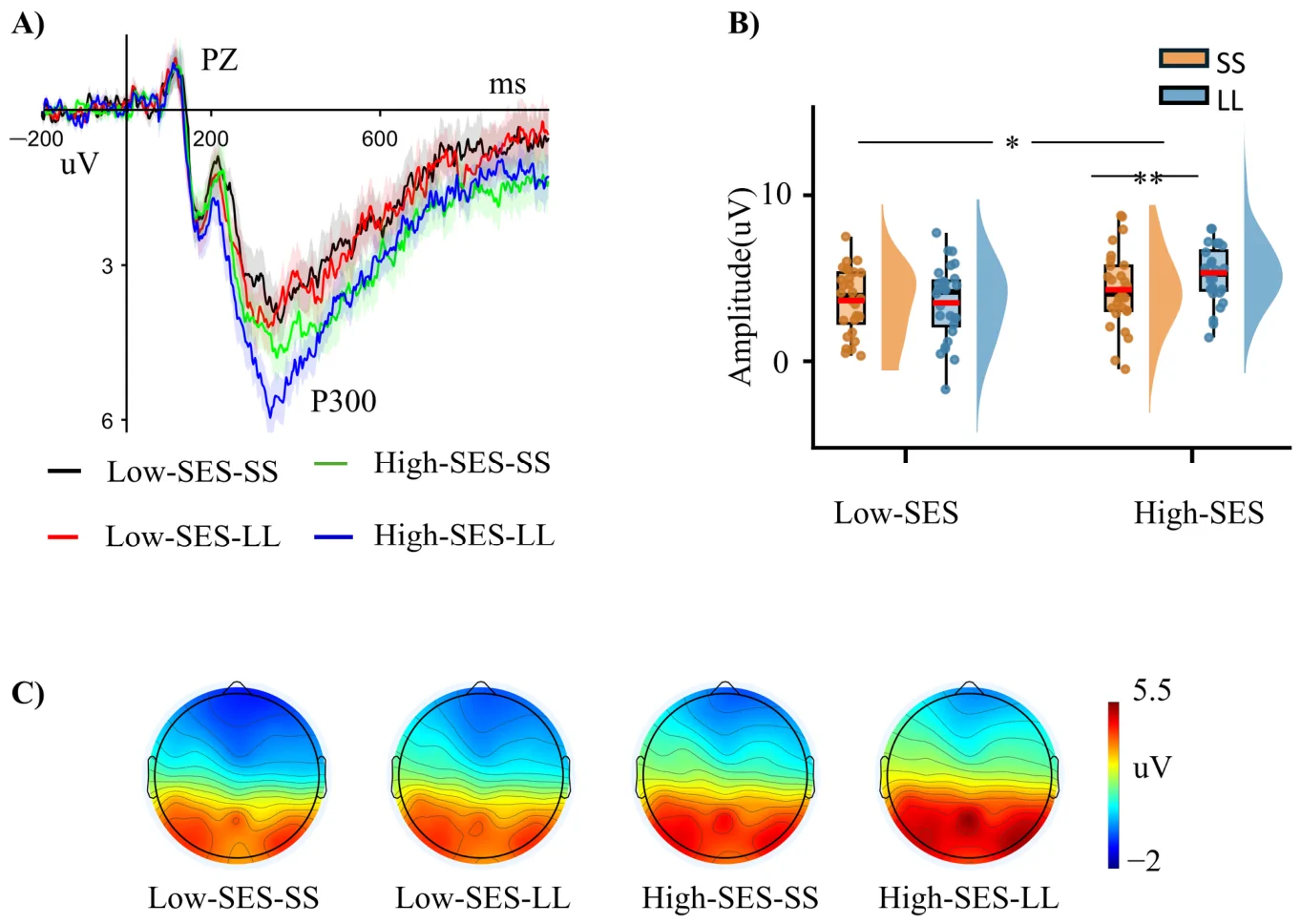
Grand-averaged ERP waveform of the P300 component (no additional filtering applied). Panel (**A**) depicts the grand-averaged P300 waveform recorded from the Pz electrode. Panel (**B**) illustrates the average P300 amplitude, and Panel (**C**) shows the scalp topography of P300. The red horizontal line indicates the mean value. “Low-SES” indicates the low-socioeconomic-status group, and “High-SES” indicates the high-socioeconomic-status group. “SS”, smaller-sooner; “LL”, larger-later. * *p* < 0.05, ** *p* < 0.01.

For the P300 amplitude, the repeated-measures ANOVA revealed a significant main effect of SES [F (1, 54) = 5.34, *p* = 0.025, $\eta_{p}^{2}$ = 0.090], indicating that the mean P300 amplitude was significantly smaller in the low-SES group than in the high-SES group. The main effect of SS/LL rewards was significant [F (1, 54) = 4.51, *p* = 0.038, $\eta_{p}^{2}$ = 0.077]. The P300 amplitude was significantly larger for the LL option than for the SS option. The interaction between SES and SS/LL rewards was significant [F (1, 54) = 7.76, *p* = 0.007, $\eta_{p}^{2}$ = 0.126]. The simple effects analysis showed that in the low-SES group, there was no significant difference in the P300 amplitude between the SS option and the LL option, F (1, 54) = 0.22, *p* = 0.643, $\eta_{p}^{2}$ = 0.004. In the high-SES group, the P300 amplitude elicited by the LL option was significantly larger than that elicited by the SS option, F (1, 54) = 12.05, *p* = 0.001, $\eta_{p}^{2}$ = 0.182. For the SS option, there was no significant difference in the P300 amplitude between the low-SES group and the high-SES group, F (1, 54) = 1.28, *p* = 0.262, $\eta_{p}^{2}$ = 0.023. For the LL option, the P300 amplitude in the high-SES group was significantly larger than that in the low-SES group, F (1, 54) = 10.17, *p* = 0.002, $\eta_{p}^{2}$ = 0.158 (Figure 6; additional details are presented in the Supplementary Materials, Table S2).

### 3.3. Discussion

Experiment 2 employed event-related potential (ERP) techniques to examine the cognitive neural mechanisms underlying the influence of SES on intertemporal choice. The behavioral results showed that the low-SES group had a higher proportion of SS choices than the high-SES group, with participants in the former also exhibiting significantly longer reaction times. The electrophysiological results revealed that the high-SES group exhibited significantly smaller P200 amplitudes, more negative N2 amplitudes, and larger P300 amplitudes than the low-SES group, providing electrophysiological evidence supporting the influence of SES on intertemporal decision-making.

## 4. General Discussion

This study investigated the associations between SES and intertemporal choice across two experiments. At the behavioral level, Experiment 1 revealed that the low-SES group had a larger ln(*k*) value than the high-SES group, with hierarchical Bayesian modeling further demonstrating that the low-SES group exhibited lower choice consistency. Experiment 2 revealed that relative to the high-SES group, individuals in the low-SES group had a higher proportion of SS choices and longer reaction times. At the electrophysiological level, Experiment 2 demonstrated that the LL option elicited more negative N2 amplitudes and greater P300 amplitudes, particularly in the high-SES group. In contrast, the low-SES group exhibited larger P200 amplitudes and smaller N2 and P300 amplitudes. Furthermore, within the low-SES group, no significant differences in P300 amplitude between SS and LL options were observed.

### 4.1. Processing Comparison Across SS/LL Rewards

This study found that the LL condition elicited more negative N2 and larger P300 amplitudes. In decision-making research, previous studies have shown that the N2 component may be associated with cognitive control processes, including response inhibition, response conflict, and error monitoring [58,59,60,64]. Present results support previous research suggesting that, compared with immediate smaller rewards, individuals’ choices of larger future rewards may be associated with a higher level of cognitive control. Neuroimaging studies may provide additional support for this interpretation, as prior research has found that cognitive control regions are more active when individuals choose future rewards rather than immediate ones [2]. Choosing an LL reward requires individuals to overcome their preference for an SS reward, thereby eliciting greater conflict [91], which may, in turn, lead to increased cognitive control [92,93,94,95,96] and more negative N2 amplitudes.

In this study, the P300 amplitude elicited by the LL option was significantly larger than that elicited by the SS option, corroborating previous research findings [62,64]. This finding is consistent with the possibility that, compared with the SS options, individuals engage more cognitive resources when faced with LL options. In intertemporal choices, future rewards are associated with greater uncertainty [85,86], increasing cognitive demands during evaluation [97]. Therefore, when selecting larger future rewards, individuals tend to allocate more cognitive resources to facilitate consideration and judgment. Additionally, previous studies have noted that in intertemporal decision-making, the greater P300 amplitude associated with non-impulsive decisions reflects more future projection and memory processing [52] or is inversely related to task difficulty [98]. The larger P300 amplitudes elicited by LL options in the present study appear to align with greater cognitive resource engagement during the evaluation of uncertain future outcomes and the formation of non-impulsive decisions that favor long-term benefits. However, it is important to acknowledge that humans’ preference for smaller-sooner (SS) rewards cannot be entirely attributed to myopia. From the perspective of ecological rationality theory [99] and the inherent uncertainty of future outcomes [100], such preferences may instead reflect an adaptive strategy that enables individuals to navigate uncertain environments in which future consequences are inherently less predictable. This perspective warrants further systematic investigation in future research.

### 4.2. Patterns of Intertemporal Decision-Making Under Low SES

The results of this study suggest that individuals with low SES have a tendency toward a preference for immediate rewards. Compared with the high-SES group, the low-SES group tended to exhibit higher ln(*k*) values in Experiment 1 and a higher proportion of SS options in Experiment 2. These converging behavioral indicators are consistent with an association between lower SES and greater delay discounting, as well as a preference for SS rewards, and are in line with previous evidence [9,11,20,21,22,23]. One possible explanation for this myopic behavior is that poverty-related daily financial concerns increase cognitive load by heightening attention to financial problems [41,44]. This cognitive load may attenuate the cognitive functions relied upon during decision-making [41,42], ultimately reducing the ability to resist immediate temptations and leading to a greater propensity for immediate rewards [25,101]. Construal Level Theory offers an alternative account by suggesting that financial scarcity is associated with a more concrete and present-focused mindset, which may undermine self-control during intertemporal decision-making and contribute to a greater tendency toward SS rewards [102].

Electrophysiological results showed that the low-SES group exhibited significantly greater P200 amplitudes than the high-SES group. Previous research in intertemporal decision-making has associated the P200 component with individuals’ tendency to engage in intuitive heuristic processing [37,56]. Within the framework of dual-system theory, the heuristic system functions automatically and rapidly, with relatively fewer cognitive resources [103]. Scarcity is frequently associated with a narrowing of attentional focus onto scarce resources, alongside an increased cognitive demand that correlates with a greater reliance on heuristic processing [104]. Moreover, research appears to link poverty to reduced working memory function [105,106,107], with rational analytic processes depending on working memory resources, while intuitive processes remain unaffected [108]. The larger P200 amplitude observed in the low-SES group during early decision-making appears consistent with rapid identification of the stimulus, pointing toward a dynamic aligned with intuitive processing.

Meanwhile, the hierarchical Bayesian analysis of the behavioral data s revealed a pattern of relatively lower choice consistency in the low-SES group than in the high-SES group. Choice consistency may be associated with decision certainty [109,110], value representation [46,111], or exploratory behavior [112]. An increasing body of research in intertemporal decision-making has associated lower consistency with more random choice behavior [6,46,49]. Previous research has also suggested that lower cognitive ability and increased cognitive load tend to be accompanied by lower choice consistency [6,48,113]. Financial scarcity is often associated with increased cognitive load [24], while lower SES is typically linked to reduced cognitive function [41,42,43], with these characteristics being related to lower choice consistency in intertemporal decision-making. These findings further align with perspectives emphasizing the roles of cognitive control and cognitive resources, offering additional support for the patterns observed in discount rates, N2, and P300 responses.

In contrast, the high-SES group showed significantly greater N2 and P300 effects in response to the LL options compared to the low-SES group. According to life history theory, high-SES individuals reared in stable environments are more likely to adopt slow strategies, placing greater value on future rewards [20,21,23] and possessing a more stable self-regulatory capacity to inhibit immediate impulses [20,22]. This finding lends preliminary electrophysiological support to the notion that the high-SES group engaged stronger cognitive control and allocated greater cognitive resources with the slow strategy.

Nevertheless, these interpretations, together with the preceding inferences, should be viewed with appropriate caution. SES is a multifaceted construct, and the behavioral and neural differences observed in the present study are likely to capture the combined influence of multiple dimensions of SES (e.g., individuals’ current real income, neighborhood characteristics, social mobility, etc.). They could also reflect the contribution of factors that were not comprehensively examined in the present study, such as executive function, chronic stress, intelligence, academic performance, etc. Future research should seek to disentangle the respective contributions of these factors while incorporating additional theoretical perspectives (e.g., depletion theory, ecological rationality, environmental uncertainty, etc.), thereby advancing a more nuanced understanding of the mechanisms linking SES to intertemporal decision-making.

### 4.3. Preferring the Present, but Not Completely Ignoring the Future

More crucially, a finding that warrants careful consideration is the absence of a significant interaction effect on the P300 component between SS and LL options in the low-SES group. Indeed, the additional inclusion Bayes Factor tends to provide moderate evidence in favor of the null interaction effect (for details on the Bayesian repeated-measures ANOVA and corresponding outputs, see the Supplementary Materials, S4). Previous research found that participants with limited budgets paid excessive attention to immediate financial-related information, such as prices [44]. However, subsequent studies did not find strong evidence supporting this tunneling phenomenon according to scarcity theory [114]. A review also noted that the evidence for this focus leading to attentional neglect of future events remains relatively weak [25]. The null P300 interaction effect offers preliminary electrophysiological evidence for a lack of significant difference in attentional bias toward present rewards versus future rewards among low-SES individuals.

In recent years, some scholars have posited that decision-making under scarcity is deliberate, to some extent, rather than thoughtless because individuals experiencing financial scarcity engage in rational trade-offs between two SS/LL rewards and make choices after careful consideration [28]. In other words, low-SES individuals do not merely focus on immediate gains while ignoring larger long-term benefits during intertemporal choice. Accordingly, this study cautiously suggests that although low-SES individuals exhibit a behavioral preference for immediate rewards, this does not necessarily imply that, at the level of cognitive processing, including cognitive resources associated with P300, they only attend to immediate gains and neglect the evaluation of future benefits. Simply equating low-SES individuals’ preference for the present with a lack of concern for or complete neglect of future rewards may represent a stereotype that warrants reconsideration [28,115]. Certainly, the present findings represent only a preliminary step toward understanding this issue, and future research is needed to further elucidate the underlying mechanisms and boundary conditions.

## 5. Conclusions

This study provided empirical evidence at both the behavioral and electrophysiological levels concerning the association between SES and intertemporal decision-making. The findings suggest that the selection of larger delayed rewards tends to be accompanied by enhanced cognitive control and the allocation of greater cognitive resources. High-SES participants exhibited a greater tendency toward such cognitive engagement patterns, alongside more future-oriented and more rational decisions. In contrast, low-SES participants appeared to rely more heavily on intuitive heuristic strategies, a pattern consistent with reduced cognitive control, lower cognitive resource allocation, a behavioral preference for smaller immediate rewards, and greater choice randomness. Furthermore, this study revealed that in the later phase of intertemporal decision-making, the low-SES group showed no significant difference in P300 compound between immediate and delayed reward options, providing novel cognitive neuroscience evidence that challenges an oversimplified interpretation of myopic decision tendencies in low-SES populations.

### Limitations and Future Directions

Several limitations of the present study should be acknowledged. First, caution is required when generalizing these findings due to the sample’s representational limitations. SES is a multidimensional construct, and consensus remains lacking regarding its measurement and deterministic factors. Although the homogeneous educational background of our sample, which comprises university students from a single city in China, helped control for potential confounders, several individual and contextual variables (e.g., childhood SES, anxiety, depression, personality traits, neighborhood characteristics, social mobility, and environmental factors) were neither fully controlled nor measured. Moreover, while participants in the low-SES group currently experience lower socioeconomic standing, their admission to higher education implies a baseline level of educational opportunities and resource availability. Thus, caution should be exercised when extending these conclusions to broader populations. Additionally, the relatively modest sample size typical of EEG experiments may constrain overall sample representativeness. Future research should encompass more diverse cohorts to enhance external validity.

Second, the cross-sectional design employed in this study precludes definitive inferences regarding causal relationships among variables. Additionally, we utilized a simplified, hypothetical intertemporal decision-making task rather than real-incentive payoffs. Although an extensive body of literature has validated the general utility and validity of hypothetical rewards in intertemporal choice paradigms [116,117], real-world intertemporal decisions in daily life are inherently more complex. Future research should consider leveraging longitudinal tracking or direct experimental manipulations to delineate causal pathways, as well as incorporating real financial incentivization schemes to bolster ecological validity.

Finally, beyond their link to the intrinsic mechanisms of intertemporal choice, it remains to be further elucidated whether EEG markers or model parameters capture additional psychological processes and whether alternative theoretical frameworks offer a more suitable account. For instance, while the low-SES group showed no significant difference between immediate and delayed options during later-stage decision processing, current evidence is insufficient to rule out variations in cognitive resource allocation. This highlights the need for more refined neurophysiological or neuroimaging indicators specifically sensitive to decision-making processes. Future studies should employ more comprehensive technical approaches from a broader perspective, while rigorously controlling extraneous variables, to further investigate these nuances.

## Data Availability

Data collection was carried out in strict compliance with the ethical guidelines and regulations of the School of Education of Hunan University of Science and Technology. The data presented in this study are available upon request from the corresponding author due to ethical and institutional regulations.

## Supplementary Materials

The following supporting information can be downloaded at https://www.mdpi.com/article/10.3390/brainsci16080879/s1, Table S1. Intertemporal Choice Task: 27 MCQ Trials; Table S2. Mean (M) Amplitudes (μV) and Standard Deviations (SD) for P200, N2, and P300 Components; S3. Supplementary Bayesian Evidence for Group Differences in Experiment 1; and S4. Bayesian Analysis of the SS–LL Interaction in the Low-SES Group on P300. References [118,119,120,121,122,123] are cited in the Supplementary Materials.

## Abbreviations

The following abbreviations are used in this manuscript:

SES	Socioeconomic Status
Low-SES	Low-Socioeconomic-Status
High-SES	High-Socioeconomic-Status
SS	Smaller-Sooner
LL	Larger-Later
ERP	Event-Related Potential
MCQ	Monetary Choice Questionnaire
