# Supplementary material for: Present-Biased Preferences Associated with Socioeconomic Status in Intertemporal Decision-Making: Evidence from Behavioral and Electroencephalographic Measures
Source: Brain Sci. 2026 Aug 18;16(8):879. doi: 10.3390/brainsci16080879 (PMC13511415; doi:10.3390/brainsci16080879)
Supplement: Supplementary file 1 [file brainsci-16-00879-s001.zip › brainsci-4454221-supplementary.pdf]

Present-Biased Preferences of Socioeconomic Status on Intertemporal Decision Making: Evidence from Behavior and Electroencephalography

Table S1. Intertemporal choice task: 27 MCQ trials

Order	Choice Trial
1	¥55 in 117 days or ¥54 today
2	¥55 today or ¥75 in 61 days
3	¥25 in 53 days or ¥19 today
4	¥85 in 7 days or ¥31 today
5	¥14 today or ¥25 in 19 days
6	¥50 in 160 days or ¥47 today
7	¥15 today or ¥35 in 13 days
8	¥25 today or ¥60 in 14 days
9	¥80 in 162 days or ¥78 today
10	¥40 today or ¥55 in 62 days
11	¥11 today or ¥30 in 7 days
12	¥75 in 119 days or ¥67 today
13	¥34 today or ¥35 in 186 days
14	¥50 in 21 days or ¥27 today
15	¥85 in 91 days or ¥69 today
16	¥49 today or ¥60 in 89 days
17	¥80 today or ¥85 in 157 days
18	¥35 in 29 days or ¥24 today
19	¥33 today or ¥80 in 14 days
20	¥28 today or ¥30 in 179 days
21	¥34 today or ¥50 in 30 days
22	¥30 in 80 days or ¥25 today
23	¥75 in 20 days or ¥41 today
24	¥54 today or ¥60 in 111 day
25	¥54 today or ¥80 in 30 days
26	¥25 in 136 days or ¥22 today
27	¥20 today or ¥55 in 7 days

Table S2. Mean (M) amplitudes (μV) and standard deviations (SD) for P200, N2, and P300 components

	Socioeconomic Status (SES)		SS/LL rewards	
	Low-SES group	High-SES group	SS rewards	LL rewards
P200	1.29 (0.37)	0.06 (0.37)	0.51 (0.29)	0.83 (0.28)
N2	- 1.44 (0.42)	- 2.69 (0.42)	- 1.72 (0.33)	- 2.40 (0.33)
P300	3.59 (0.38)	4.82 (0.38)	3.98 (0.29)	4.42 (0.28)

Note: Low-SES group (*N*=28) and high-SES group (*N*=28). Values are mean amplitudes (μV) averaged across selected electrodes. Standard deviations appear in parentheses.

S3. Supplementary Bayesian Evidence for Group Differences in Experiment 1

To complement the hierarchical Bayesian parameter estimation reported in the main text, we additionally quantified the evidence for the presence of group differences using Bayes factors. Whereas the main analyses focused on posterior parameter estimates and their corresponding 95% highest density intervals (HDIs), Bayes factors provide a direct measure of the relative evidence for the alternative hypothesis (H_1) versus the null hypothesis (H_0). These are computed as the ratio of the integral of the posterior difference distribution from 0 to ∞ versus the integral from 0 to -∞. Using common criteria (Beard, Dienes, Muirhead, & West, 2016; Jeffreys, 1998; Lee & Wagenmakers, 2013):

BF < 1/100: overwhelming evidence in favor of H0;
1/100–1/30: very strong evidence in favor of H0;
1/30–1/10: strong evidence in favor of H0;
1/10–1/3: moderate evidence in favor of H0;
1/3–1: anecdotal evidence in favor of H0;
1–3: anecdotal evidence in favor of H1;
3–10: moderate evidence in favor of H1;
10–30: strong evidence in favor of H1;
30–100: very strong evidence in favor of H1;
BF > 100: extreme evidence in favor of H1.

The Bayes factor analysis yielded **BF_{10} = 10.25** and the corresponding reciprocal value **BF_{01} = 0.097** for discounting rate, **BF_{10} = 13.92** and the corresponding reciprocal value **BF_{01} = 0.071** for choice consistency. These results provide strong evidence in favor of a difference between the low-SES group and the high-SES group.

S4 Bayesian Analysis of the SS–LL Interaction in the Low-SES Group on P300

In addition to frequentist analyses, Bayesian repeated-measures ANOVAs were conducted in JASP (version 0.95.0; JASP Team 2024) with default priors for SS rewards vs. LL rewards in the low-SES group with P300 compound. For the main effect of SS/LL rewards, Bayesian inclusion factors (BF_incl) were estimated, which compare the predictive performance between models containing the effect and equivalent models without that effect. In practical terms, BF_incl quantifies how much the probabilities in favor of an effect being present change after observing the data, compared to the prior probability (Rouder, Morey, Speckman, & Province, 2012; van Doorn et al., 2021).

For SS rewards vs. LL rewards in the low-SES group,

P(incl | data) = 0.31;

P(excl | data) = 0.69;

BF_incl = 0.29.

The inclusion of the Bayes factor provided moderate evidence against the interaction effect of P300.

Beard, E., Dienes, Z., Muirhead, C., & West, R. (2016). Using Bayes factors for testing hypotheses about intervention effectiveness in addictions research. *Addiction, 111*(12), 2230-2247. doi:10.1111/add.13501

Jeffreys, H. (1998). *Theory of Probability*. Oxford University Press.

JASP Team. 2024. Version 0.95.0, https://jasp-stats.org/download.

Lee, M. D., & Wagenmakers, E.-J. (2013). *Bayesian cognitive modeling: A practical course*. New York, NY, US: Cambridge University Press.

Rouder, J. N., Morey, R. D., Speckman, P. L., & Province, J. M. (2012). Default Bayes factors for ANOVA designs. *Journal of Mathematical Psychology, 56*(5), 356-374. doi:10.1016/j.jmp.2012.08.001

van Doorn, J., van den Bergh, D., Böhm, U., Dablander, F., Derks, K., Draws, T., . . . Wagenmakers, E. J. (2021). The JASP guidelines for conducting and reporting a Bayesian analysis. *Psychon Bull Rev, 28*(3), 813-826. doi:10.3758/s13423-020-01798-5
